# Identification and Fine Mapping of a White Husk Gene in Barley (*Hordeum vulgare* L.)

**DOI:** 10.1371/journal.pone.0152128

**Published:** 2016-03-30

**Authors:** Wei Hua, Xiao-Qi Zhang, Jinghuan Zhu, Yi Shang, Junmei Wang, Qiaojun Jia, Qisen Zhang, Jianming Yang, Chengdao Li

**Affiliations:** 1 Zhejiang Academy of Agricultural Sciences, Hangzhou, 310021, China; 2 Western Barley Genetics Alliance, Murdoch University, Murdoch, WA 6150, Australia; 3 Australian Export Grain Innovation Centre, South Perth, WA 6151, Australia; University of Tasmania, AUSTRALIA

## Abstract

Barley is the only crop in the Poaceae family with adhering husks at maturity. The color of husk at barely development stage could influence the agronomic traits and malting qualities of grains. A barley mutant with a white husk was discovered from the malting barley cultivar Supi 3 and designated *wh* (white husk). Morphological changes and the genetics of white husk barley were investigated. Husks of the mutant were white at the heading and flowering stages but yellowed at maturity. The diastatic power and α-amino nitrogen contents also significantly increased in *wh* mutant. Transmission electron microscopy examination showed abnormal chloroplast development in the mutant. Genetic analysis of F_2_ and BC_1_F_1_ populations developed from a cross of *wh* and Yangnongpi 5 (green husk) showed that the white husk was controlled by a single recessive gene (*wh*). The *wh* gene was initially mapped between 49.64 and 51.77 cM on chromosome 3H, which is syntenic with rice chromosome 1 where a white husk gene *wlp1* has been isolated. The barley orthologous gene of *wlp1* was sequenced from both parents and a 688 bp deletion identified in the *wh* mutant. We further fine-mapped the *wh* gene between SSR markers *Bmac0067* and *Bmag0508a* with distances of 0.36 cM and 0.27 cM in an F_2_ population with 1115 individuals of white husk. However, the *wlp1* orthologous gene was mapped outside the interval. New candidate genes were identified based on the barley genome sequence.

## Introduction

Barley (*Hordeum vulgare* L., 2n = 2H = 14) is the only Poaceae family crops with its caryopsis adhering to husk, which provides economically important source of human and animal nutrition and underpins the malting and brewing industries. Barley also can be used as a genetic model species for Triticeae genomics [1]. In general, the husk is green, which not only protects the flower and grain from pathogens, insects, and external environments, but also is the essential photosynthetic organ to accumulate dry matter of grain [2,3]. Studies have shown that photosynthesis in husk occurs via C3 and C4 pathways, and they can provide carbohydrates and amino acids for grain growth [4,5]. Therefore, the changes in husk color could affect on the compositions of the grain and the yield of barley.

The white husk is a special type of husk color variation. In barley, a white husk mutant was first described in 1958 as a natural mutant from an unknown variety [6]. Genetic analysis of this mutant identified that the white husk characteristic is controlled by a recessive nuclear gene named *alm*, located on the short arm of chromosome 3H using a full set of morphological traits as markers [7–9]. Linkage maps, including the *alm* gene, have been constructed on the basis of morphological and molecular markers [10]. However, these maps exhibit low density near the *alm* gene. In rice, five white husk genes have been identified and they are *wp1*[11], *wp2*[12], *wp(t)*[13], *wslwp*[14], and *wlp1*[15]. To date, only *wlp1* has been cloned and one base substitution of C to T in the coding region of chloroplast ribosome L13 protein could result in white husk and leaf under low temperature[15].

The recent release of the barley draft genome assembly [16] provides a resource for pursuing the target gene, however, the precise genomic sequence is still not available. Early molecular genetic studies, based on sets of cloned DNA probes hybridizing to genomic DNAs of different species, have further developed our understanding of the conservation of gene synteny within the grass family, and lead to the concept of alignment of different grass genomes at the macro level [17–20]. Since completing the draft rice genome sequence [21], rice has been successfully used as a model crop to isolate functional genes in barley based on syntenic cloning [22–24]. In this study we report the discovery of a white husk barley mutant, its husk morphological features and genetics. The husk gene was initially mapped to chromosome 3H and a candidate gene identified using the syntenic cloning approach. Further fine-mapping identified that the rice orthologous gene is not the functional gene for the mutant in barley. These results will pave the way for map-based cloning of the target gene and unraveling the molecular mechanisms of the mutant. The white husk gene/trait will be utilized as a visualized marker in barley molecular breeding in future.

## Materials and Methods

### Plant materials

A mutant with a white husk at the heading and flowering stages was identified from a commercial malting barley variety Supi 3 in the field of Jiangsu Province of China in 2006 and designated *wh* (white husk). The mutant *wh* was crossed with a green husk variety Yangnongpi 5 to obtain F_1_ hybrid seeds in 2009. The F_1_ was then crossed with *wh* and Yangnongpi 5, respectively to produce BC_1_F_1_ progenies. The F_2_ populations, BC_1_F_1_ progenies, their F_1_ and parents were planted in the field at Zhejiang Academy of Agricultural Sciences, Hangzhou, China to observe the husk color of individuals at the heading stage, and then to analyze inheritance of the white husk.

Two hundred and twenty-eight individuals with white husks from the *wh*/Yangnongpi 5 F_2_ population were used for preliminarily mapping of the target gene. One thousand one hundred and fifteen individual plants with white husks of an enlarged F_2_ population (4657 individuals) were used to fine-map the white husk gene.

### Agronomic traits and malting quality analysis

Agronomic traits (plant height, 1000-grain weight and maturity) of *wh* and its wild-type Supi 3 were investigated as described by Zhang and Liu [25]. Micro-malting and malting quality analysis was conducted in the laboratory of the National Barley Improvement Centre (Hangzhou, China) using 120 g of grain from the sieving fraction >2.2 mm. The malting process and determination of malting quality such as malt extract, viscosity, diastatic power, α-amino nitrogen and kolbach index were performed as described by Wang et al. [26].

### Morphological analyses of white husks by SEM and TEM

The lemmas of *wh* and its wild-type Supi 3 at the booting and heading stages were obtained, cut into small pieces (2 mm) and fixed in 2.5% glutaraldehyde (v/v) in 100 mM phosphate buffer (PBS, pH 7.0) for 6 to 8 h. The samples were washed three times with the same PBS, post-fixed in 1% osmium tetroxide for 1 h, and dehydrated with an ethanol series. Some of the samples were then treated for scanning electron microscopy (SEM), with the remaining samples used for transmission electron microscopy (TEM). SEM samples were initially transferred to an alcohol and iso-amyl acetate mixture (v:v = 1:1) for approximately 30 min and then to pure iso-amyl acetate for approximately 1 h. The specimens were dehydrated using a Hitachi Model HCP-2 critical point dryer with liquid CO_2_. The dehydrated specimens were coated with gold-palladium and observed using a Philips Model TM-1000 SEM. TEM samples were treated with gradient ethanol concentrations and transferred to absolute acetone for 20 min. The specimens were then placed in a 1:1 absolute acetone and Spurr resin mixture for 1 h at room temperature, before being transferred to a 1:3 mixture of absolute acetone and resin for 3 h. The resulting sample was transferred to the final Spurr resin mixture and stored overnight. The specimen was stained with uranyl acetate and alkaline lead citrate, and ultrathin sections (80 nm) were prepared. These sections were examined using a TEM (Hitachi H-7650, Japan).

### Genomic DNA extraction

Seedling leaves from F_1_, F_2_, and parent plants were collected and frozen in liquid nitrogen for DNA extraction. The cetyltrimethylammonium bromide (CTAB) method was used to extract genomic DNA from 0.6 to 1.0 g of young leaf tissue [27]. The quality and concentration of DNA was estimated using a NanoDrop ND-2000 spectrophotometer (Thermo Scientific, Walthman, MA, USA). DNA samples were diluted with double-distilled H_2_O to obtain a final concentration of 100 ng/μL and stored at –20°C. Bulk segregant analysis was initially used to map the *wh* gene. Two DNA pools were assembled using equal amounts of DNA from 10 F_2_ plants with white or green husks.

### SSR marker selection

A total of 1046 SSR markers [28–32] distributed in the seven chromosomes of barley were used to screen for polymorphism between the two parental lines (*wh* and Yangnongpi 5). Polymorphic SSR markers were first used to analyze the two DNA pools. The polymorphic markers between the two bulks were used to determine the genotype of individuals from F_2_ plants with white husks.

Polymerase chain reaction (PCR) was performed in a final reaction volume of 10 μL containing 1 μL of 100 ng/μL genomic DNA, 1 μL of 10 pmol/L solution of forward and reverse primers, 3 μL of sterile ddH_2_O, and 5 μL of 2× *Taq* Master Mix (GeneSolution, Shanghai, China). PCR products were separated on 8% non-denaturing polyacrylamide gels and visualized by silver staining.

### Data analysis

Chi-squared (χ^2^) tests for goodness-of-fit were performed to evaluate the deviations of observed data from theoretically-expected segregation ratios. Polymorphic molecular markers and the white husk gene were subjected to linkage analysis using JoinMap 3.0 software with LOD threshold of 4 [33]. Recombination frequencies were converted to map distances in centiMorgans (cM) by the Kosambi function [34].

### Comparative genomic analysis

SSR markers flanking the target gene were first integrated into the barley consensus map [35]. Sequences of the molecular markers in the regions were used to search the barley genomic database (http://webblast.ipk-gatersleben.de/barley/viroblast.php) to identify corresponding barley contigs. The markers flanking the white panicle genes in rice were mapped to the corresponding physical map (http://archive.gramene.org/markers/), and the genes around these markers identified on the Rice Genome Browser website (http://rice.plantbiology.msu.edu/cgi-bin/gbrowse/rice/). For collinearity analysis of barley and rice, the sequences of these rice genes were used to blast barley genomic databases and to find their orthologous loci. Where several significant hits were found, only the best hit was adopted.

### Testing a *wlp1* barley orthologous gene

According to the synteny between barley and rice, the sequence of the *wlp1* gene (LOC_Os01g0749200) in rice was used to search orthologous genes in the barley genomic database (http://webblast.ipk-gatersleben.de/barley/) to identify a Morex_Contig_48768 (MC_48768). The orthologous gene was sequenced in *wh*, Supi 3 and Yangnongpi 5 with the following primers: forward primer (*Hvwlp1*-F1), 5’-TCCTCCTCCCTCCTTCTATCCC, and reverse primer (*Hvwlp1*-R1), 5’-GAGCAGCCTGCATCATCTCACT, to amplify ORF (open reading frame) of the gene; forward primer (*Hvwlp1p*-F1), 5’- TGCCATCAAAGGAGGATAGAAA, and reverse primer (*Hvwlp1p*-R1), GGGGAACGAGTGCGGAAAGAGG, to amplify the promoter region.

A PCR reaction was conducted using a KOD-FX kit (TOYOBO, Japan) comprising 6 μL of 2 mM dNTP, 30 μL of 2× buffer, 1.2 μL of 1 U/μL KOD-FX polymerase, 3.6 μL of 10 pmol/L solution of forward and reverse primers, 6 μL of 100 ng/μL genomic DNA, in a final volume of 60 μL with sterile distilled water. PCR cycling conditions consisted of an initial denaturation setup of 94°C for 4 min, followed by 35 cycles of 94°C for 30 s, 55°C for 30 s, 72°C for 2 min 30 s, and a final extension cycle at 72°C for 10 min. At least two independent PCR products were sequenced by BGI Tech Solutions Co., Ltd (Shenzhen, China).

## Results

### Discovery of a white husk barley

**Fig 1:**
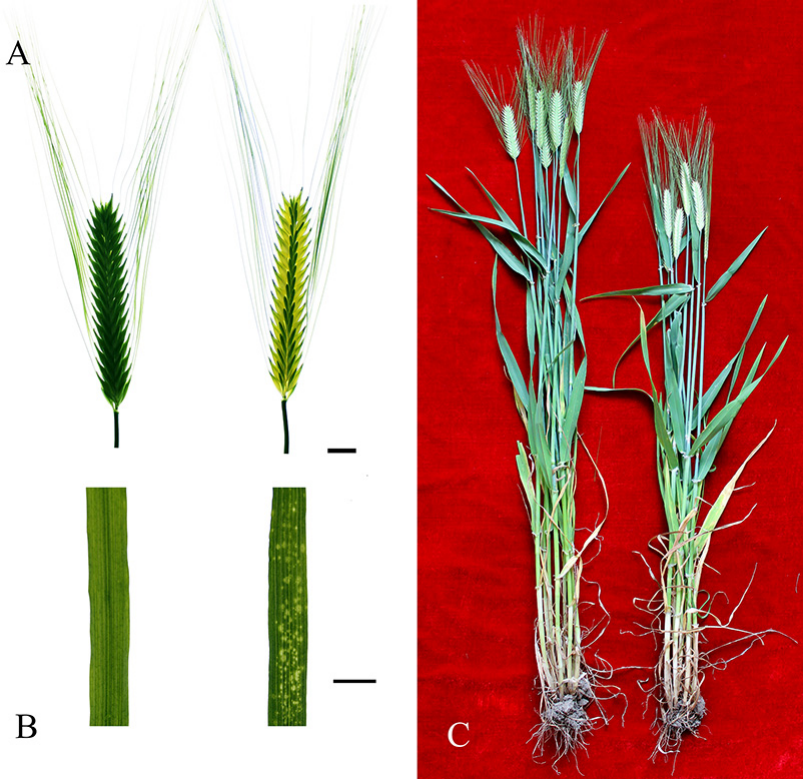
Phenotype of wild-type Supi 3 and mutant *wh*. (A) spikes, (B) leaf, and (C) whole plant in the filling stage. Supi 3 on the left and *wh* on the right. The bar represents 1 cm.

In 2006, a mutant was discovered in a field of malting barley cultivar Supi 3, a leading cultivar in the Jiangsu Province, China. The husks in the mutant were colorless at the heading and flowering stages (Fig 1A) and became yellow but slightly brighter than Supi 3 in the mature grains. Husk color of mature grains of Supi 3 and *wh* were measured with a Konica Minolta CM-700d over two consecutive years; the mutant values for Min L (brightness) were 2.4 units higher than Supi 3 (Table 1), indicating brighter grains. Multi-year and -location experiments showed that the white husk trait is stably inherited.

**Table 1:** Characterization of *wh* grains. Husks in mature grains of *wh* were much brighter, as diastatic power and α-amino nitrogen contents significantly increased, even though malt extract decreased slightly.

Year	Material	L values	Viscosity (mPa·s)	Malt extract (%)	Diastatic power (W·K)	α-amino nitrogen (mg/100 g)	Kolbach index (%)
2011	supi3	60.7	0.942	77.49	449.7	150.30	35.26
	*wh*	63.14	0.945	72.81*	542.5**	219.60**	37.33
2012	supi3	59.21	1.034	80.10	378.5	192.08	46.24
	*wh*	61.63	0.991**	77.67**	513.9**	256.62**	48.61

* and ** signification at P = 0.05 and 0.01, respectively.

In contrast to the wild-type barley, *wh* had yellow spots on its leaves during vegetative growth (Fig 1B). Plant height in *wh* was 7–14% shorter than its wild type in two consecutive years (Fig 1C). The 1000-grain weight of *wh* was 82–87% and its maturation period was also 5–7 d earlier than its wild-type barley. Furthermore, the diastatic power and α-amino nitrogen contents were much higher in the *wh* than in Supi 3 grains (Table 1).

**Fig 2:**
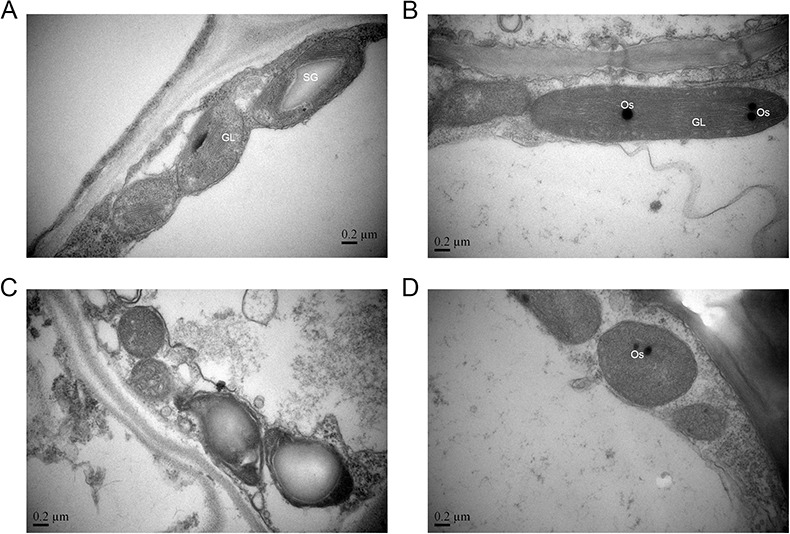
TEM micrographs of the chloroplast of spike lemma of wild-type Supi 3 (top) and mutant *wh* (bottom) barley at different growing stages. (A, C) booting stage, and (B, D) heading stage. GL: granum lamellae; Os: osmiophilic plastolobuli; SG: starch grain.

Scanning electron microscopy showed that mutant grains had more dense trichomes than wild-type barley grains. Yet, they were not taller or plumper; projections could not be formed on top in *wh* lemma surface (S1 Fig). Differences were evident in morphological characteristics and internal structure of the chloroplast between wild-type and mutant lemma at the booting and heading stages (Fig 2). The wild type had oblong chloroplasts with a dense internal structure, and normal growth at these two stages. The laminated structure of the thylakoids was regular (Fig 2A and 2B). Mutant chloroplasts had numerous empty vesicle-like structures at the booting stage, and abnormal growth. The laminated structure of the thylakoids was dispersed, and the chloroplasts produced cysts. At the heading stage, no laminated structure was observed and the membrane structure of the chloroplast was destroyed. Thus, serious growth defects were observed in the mutant barley chloroplasts (Fig 2C and 2D).

### Genetic analysis of white husk barley

At the heading stage, *wh* husks were white, while husks of Yangnongpi 5 are normally green. When *wh* and Yangnongpi 5 were reciprocally crossed, F_1_ generation plants had green husks. Thus, the white husk trait for *wh* is controlled by a recessive nuclear gene. Among the 970 F_2_ plants of *wh*/Yangnongpi 5, 742 single plants had normal green husks and 228 had white husks. The segregation ratio fits the 3:1 model (χ^2^_3:1_ = 1.08, P > 0.3). Of the 93 BC_1_F_1_ plants of *wh*/Yangnongpi 5//*wh* backcrossing, 50 single plants had normal green husks, and 43 had white husks; the segregation ratio was 1:1 (χ^2^_1:1_ = 0.39, P > 0.5). All 97 BC_1_F_1_ single plants from the *wh*/Yangnongpi 5//Yangnongpi 5 backcrossing had normal green husks. These results confirm that the white husk trait is controlled by a recessive nuclear gene.

### Mapping the white husk gene *wh*

Nine hundred and forty-four pairs of the published SSR primers synthesized in our laboratory were screened for polymorphism between the parents *wh* and Yangnongpi 5. Among these primer pairs, 95 were polymorphic between parents. The polymorphic SSR markers were used to test for polymorphism in the two DNA pools. Five SSR markers—namely *Bmac0209*, *Bmag0138*, *Bmac0871*, *Bmag0508a*, and *Bmag0122*—located on chromosome 3H were polymorphic between the two DNA pools. The five polymorphic markers were used to analyze the 228 individuals with white husks from the F_2_ population. Linkage analysis showed that *wh* was located on the short arm of chromosome 3H.

### Collinearity analysis of the white husk genomic region in barley and rice

**Fig 3:**
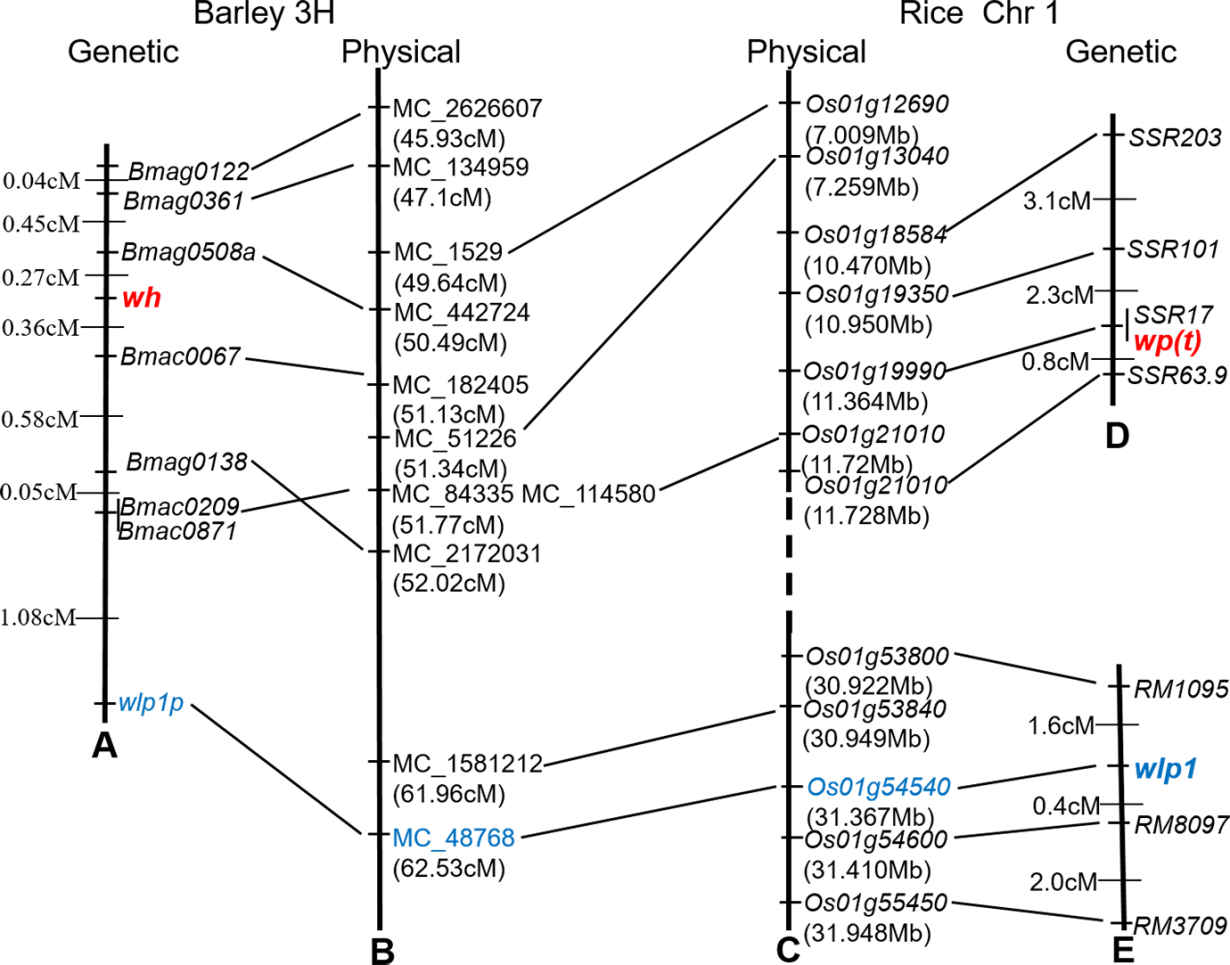
Linkage map of *wh* and collinearity analysis with rice chromosome 1H. (A) genetic linkage map of *wh*, (B) physical map covering *wh* in barley chromosome 3H, (C) physical map containing *wp(t)* and *wlp1* in rice chromosome 1, (D) genetic map of *wp(t)* according to [13], and (E) genetic map of *wlp1* according to [15]. The numbers on the left indicate genetic distances (cM) between adjacent loci, and the numbers in brackets indicate physical positions. The dotted line indicates the discontinuity in the rice physical map. All positions are not to scale.

All markers (S1 Table) in the interval with sequences were used to search for corresponding barley contigs (http://webblast.ipk-gatersleben.de/barley/). The markers were mapped to an interval between 45.93 and 51.77 cM (Fig 3A and 3B) on chromosome 3H of the POPSEQ map [36]. Sequences of all barley genes in the interval were retrieved and used to BLAST search the syntenic rice genes. The genomic region in barley chromosome 3H showed good synteny with rice chromosome 1 with conserved gene order (Fig 3).

Of the five white panicle genes in rice [11–15], *wp(t)* and *wlp1*—mapped to rice chromosome 1—were located in the syntenic region of the barley mutant gene *wh* on chromosome 3H.

### Isolation of the orthologous gene of *wlp1* in *wh* and its wild type

Of the five white panicle genes in rice, *wlp1* was only one cloned and its full sequence was 2402 bp, which included four exons and three introns. By the sequence blast, the barley contig MC_48768 shared 92% identity with the genomic DNA sequence of *wlp1*. MC_48768 contained a gene model (MLOC_64398.1) annotated as 50S ribosomal protein L13 (http://apex.ipk-gatersleben.de/apex/f?p=284:20:0::NO::P20_GENE_NAME:MLOC_64398.1). In order to establish whether this gene was *wh*, we designed two forward primers at 1781 bp and 119 bp upstream of the initiation codon and two reverse primers at 171 bp and 2364 bp downstream of the initiation codon to amplify, respectively, the promoter and ORF of the orthologous gene of *wlp1* in *wh* and its wild type. The PCR products were 1953 bp and 2484 bp, respectively (S2 Fig). No difference in DNA sequences was identified between *wh* and its wild type (S3 Fig). Sequence analysis identified a 688 bp deletion in the *wh* mutant compared to Yangnongpi 5 (S2 Fig). The polymorphism made it possible to map the *wlp1* orthologous gene in the *wh*/Yangnongpi 5 population (Fig 3).

### Fine-mapping the *wh* gene

To fine-map the *wh* gene, we expanded the F_2_ population of *wh*/Yangnongpi 5 to 4657 individual plants and used 1115 individuals with white husk of them to test the five polymorphic markers in the preliminary mapping results. Meanwhile, to identify more molecular markers linked with this gene, we screened an additional 102 published SSR primer pairs and EST-SSR primers on chromosome 3HS. Two additional markers *Bmac0067* and *Bmag0361* were linked with the *wh* gene, and the *wh* gene was mapped between *Bmac0067* and *Bmag0508a* markers with genetic distances from *wh* of 0.36 and 0.27 cM, respectively (S2 Table) (Fig 3A). However, the *wlp1* orthologous gene was mapped 2.07 cM from the *wh* gene. The fine-mapping results demonstrated that the barley *wh* is not the rice *wlp1* orthologous gene. Further analysis indicated that the rice genomic region around *wp(t)* between 7009 kb and 11720 kb was co-linear with the barley genomic region between MC_1529 and MC_114580, which is more than likely the orthologous gene for *wh* (Fig 3). However, no functional gene has been identified for the *wp(t)* locus.

## Discussion

In this study, we reported a white husk mutant *wh* from the malting barley cultivar, Supi 3. By SEM and TEM observations, we found that the external and internal structures of the lemma in *wh* differed from the wild type. Thus, the white husk of *wh* may be due to abnormal chloroplast development. As compared to Supi 3, the white husk in *wh* mutant lead to the reduction of 1000-grain weight and malt extract, and the increase of the diastatic power and α-amino nitrogen contents. It was probably due to that the abnormal chloroplast metabolism in white husk influenced the photosynthesis of husks, which then had some impacts on the weight and malting qualities of grains. In addition, the plant height was significantly shorter and the maturation period was about 7 days earlier in *wh*. It was unknown whether these phenomena were caused by white husk. However, the similar cases have also reported in other mutants. For example, a barley stage green-revertible albino mutant *whs18* was reported, in which the plant height was obviously shorter and its heading date was about 8 days later than its wild type [37].

We mapped the white husk gene *wh* between *Bmac0067* and *Bmag0508a* on the barley chromosome 3H. A spontaneous mutant *Russia 82* was reported with albino lemma (*alm*) gene in 1958 [6]. Costa et al. [10] used the albino lemma trait (*alm*) as an anchor marker to construct a molecular linkage map and found that the AFLP marker E38M62-81 co-segregated with the *alm* locus. In our study, we tested the polymorphism between the parents using the marker *E38M62-81* but no polymorphism was found in our population. More research is needed to determine whether *alm* and *wh* are allelic.

In previous studies, many leaf color mutants have been reported, such as albino, viridis and xantha [38]. Of these, the white leaf is an extreme trait that is unable to trap light energy. Therefore, most white leaf mutants cannot survive. Although extensive attention has been paid to albinism, the mechanism of mutation and the responsible loci are not fully understood at the molecular level. In *Arabidopsis*, four genes—*CLA1* [39,40], *ALB3* [41], *ALB4* [42] and *CLB6* [43]—which all result in the albino phenotype were isolated; these genes encode 1-deoxyxylulose 5-phosphate synthase, a chloroplast protein, thylakoid membrane protein and 1-hydroxy-2-methyl-butneyl 4-diphosphate reductase, respectively. *CLA1* and *CLB6* play important roles in the methy-D-erythritol 4-phosphate pathway. *ALB3* may have an important function in the assembly of a chloroplast enzyme complex while *ALB4* is involved in proper chloroplast biogenesis. In rice, Gothandam et al. [44] transferred the antisense *OsPPR1* gene and all transgenic plants were albino, suggesting that the PPR protein is required for chloroplast biogenesis. Su et al. [45] reported a *ysa* mutant which appeared albino before the three-leaf stage, but gradually turned green from the four-leaf stage. The *ysa* gene was isolated using map-based cloning and shown to encode a new member of the superfamily of PPR proteins. In barley, Qin et al. [37]reported that a stage green-revertible albino mutant *whs18*, which could be caused by the substitution from C to A in the third exon of fructokinase-1-like gene generated a premature stop codon in *whs18*. The husks are unique organs in the Poaceae that protect the inner floral organs and kernels from environmental stresses; they are also photosynthetic organs that supply carbohydrates and amino acids for grain growth and development [46,47]. Song et al. [15] reported a *wlp1* (white leaf and panicles 1) mutant in rice with albino early seedling leaves, and albino immature panicles at the heading stage. These abnormal leaf and husk color was induced by low temperature. The *wlp1* gene encodes a 50S ribosome L13 protein which is required for normal chloroplast development, and is the only cloned gene related to the white panicle trait in the Poaceae. The gene was located in the syntenic region of the *wh* gene from the initial comparative analysis. It would be logical to speculate that the *wlp1* is the orthologous gene of barley *wh*, as a large deletion was identified in the promoter region between *wh* and Yangnongpi 5 (S2 Fig). However, fine-mapping excluded this gene as the functional gene for the *wh* mutant. Thus, it should be worth to revisit some genes identified through syntenic map. In some cases, fine-mapping may be required.

We mapped the *wh* gene into a 0.6 cM interval (Fig 3). There are 40 high confident genes (S3 Table) in this interval based on the POPSEQ [36]. The mutant *wh* showed abnormal chloroplast development (Fig 2), but no obvious gene has been identified in chloroplast development in this interval. The four genes encoding zinc finger family protein, histone H2B, nucleic acid-binding OB-fold-like protein, and ATP-dependent RNA helicase may be reasonable candidates for the *wh* gene. Further research is required to identify the functional gene for the *wh*.

## Supporting Information

S1 Fig SEM micrographs of lemma under different magnifications at the booting stage. (A, B) wild-type lemma with trichomes on a surface, and (C, D) mutant lemma with trichomes on a surface. The bars in A, C represent 200 μm and the bars in B, D represent 30 μm.
(PPTX)

S2 Fig **The sketch map of the *wlp1* orthologous gene (A) and the information of *wlp1p* marker (B).** The numbers in the pane represent the size of exons. + and—represent the downstream and upstream of the initiation codon, respectively, and +1 represents the A position of the initiation codon ATG. The size of the fragments is not to scale.
(DOCX)

S3 Fig Sequence alignment of the *wlp1* orthologous gene in three barley varieties. (EMF)

S1 Table All marker in the target region. (XLSX)

S2 Table The genotyping information for recombinants in F_2_ population of *wh*/Yangnongpi 5. (XLSX)

S3 Table All genes in the target region based on the POPSEQ. (XLSX)
