# Supplementary figures and images for: Identification and Fine Mapping of a White Husk Gene in Barley (Hordeum vulgare L.)
Source: PLoS One. 2016 Mar 30;11(3):e0152128. doi: 10.1371/journal.pone.0152128 (PMC4814061; doi:10.1371/journal.pone.0152128)

## Slide 1
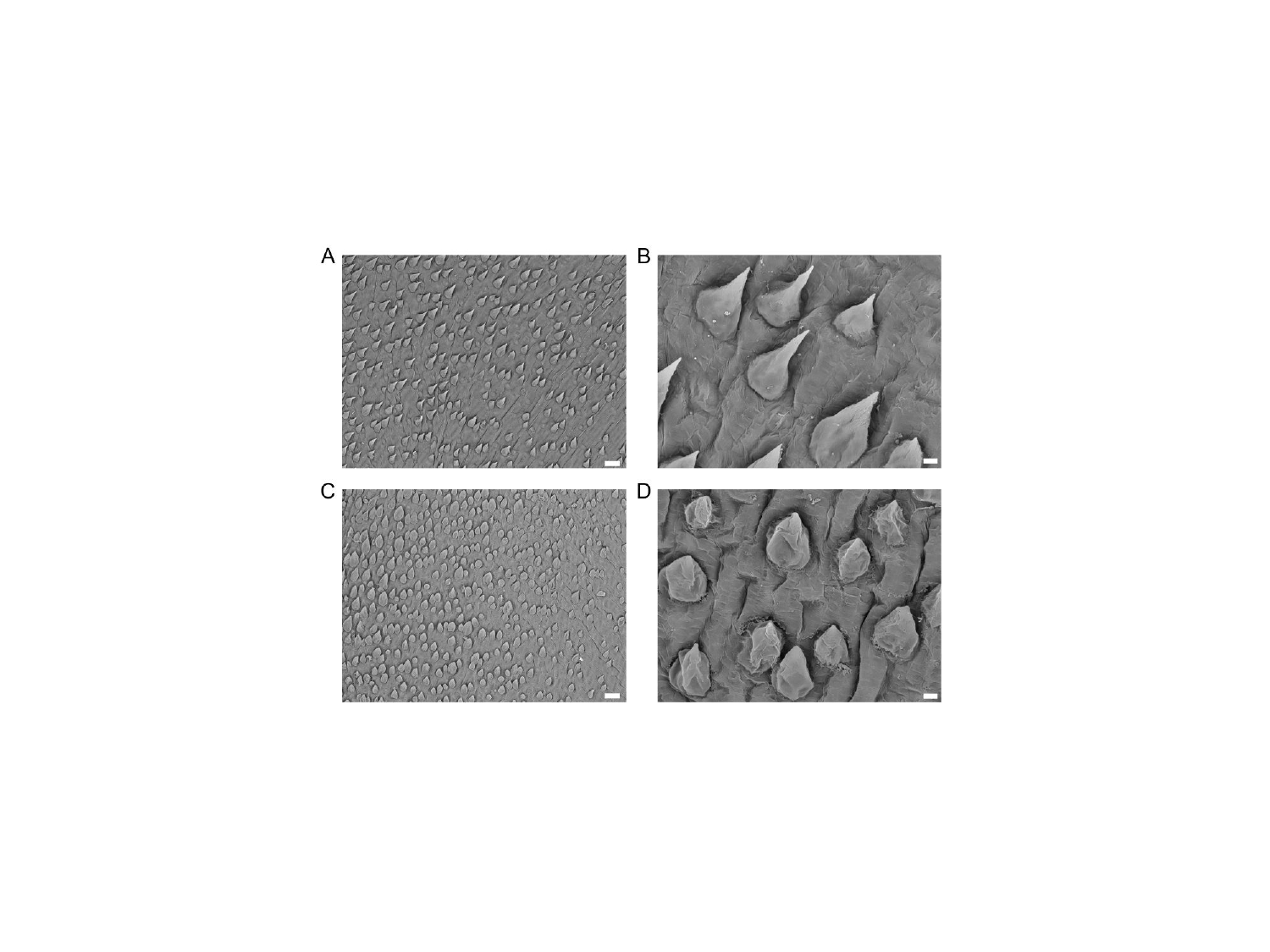

Supplement: S1 Fig — (A, B) wild-type lemma with trichomes on a surface, and (C, D) mutant lemma with trichomes on a surface. The bars in A, C represent 200 μm and the bars in B, D represent 30 μm. (PPTX) [file pone.0152128.s001.pptx]

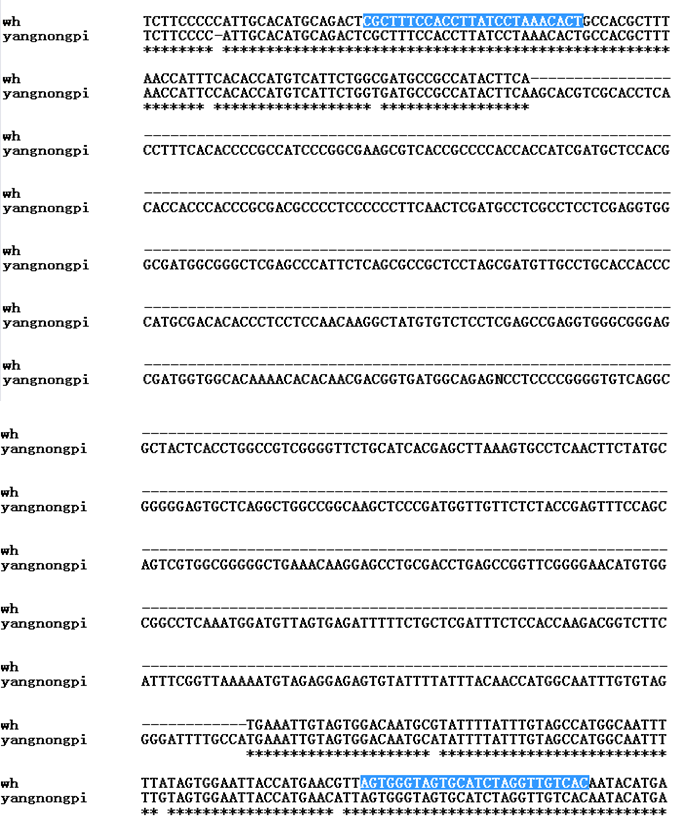


蓝色背景的序列为设计的Indel引物序列

Supplement: S2 Fig — The sketch map of the wlp1 orthologous gene (A) and the information of wlp1p marker (B). The numbers in the pane represent the size of exons. + and—represent the downstream and upstream of the initiation codon, respectively, and +1 represents the A position of the initiation codon ATG. The size of the fragments is not to scale. (DOCX) [file pone.0152128.s002.docx]
